# Etherization in Surgery, with Surgical Remarks

**Published:** 1849-09

**Authors:** 


ART III.—*Etherization, with Surgical Remarks.*—By JOHN C. WARREN, M. D., Emeritus Prof of Anatomy and Surgery, in the University at Cambridge, &c. &c. Boston, Ticknor & Co. 1848. pp. 100, 12 mo. (From the Publishers.)

This little volume is very interesting, and constitutes a very valuable portion of the history of anæsthetic agents. Its author was among the first surgeons who resorted to the use of these agents, which were first discovered in the city in which he resides. From Boston the use of ether and kindred substances, spread to all parts of the civilized world; and although violently opposed in some quarters, it may now be regarded as settled, that no surgeon is justified, when about performing a severe operation, in witholding these agents, unless he should be of opinion that some peculiar circumstance connected with the case, might render the administration unsafe.

As this work was furnished us at a late date; and as since its publication much new light has been shed upon this interesting and important subject, and, moreover, as we design giving our readers at some future time, a full view of the whole subject; we forego any furthur remarks on this volume, contenting ourselves with quoting the general conclusions at which the distinguished author arrived, concerning the use of anæsthetic agents. M

GENERAL CONCLUSIONS.---The views we have taken on this subject suggest the following inferences.

1st. Inhalation of ether produces insensibility to pain.

2d. Ethereal insensibility, judiciously effected is not followed by any dangerous consequenccs.

3d. Its administration is easy, and requires but a few minutes.

4th. Individual of all ages may be safely etherized.

5th. Surgical operations may be done under the effects of ether, which could not be done withou.

6th. The shock of the nervous system is diminished.

7th. The use of ether has greatly increased the number of successful operations, by encouraging a resort to them at an earlier period of disease.

8th. The use of the sponge is more safe than that of any apparatus.

9th. The existence of chronic pulmonary disease rarely forms an objection to etherization.

10th. Etherization may be employed as a substitute for narcotics.

11th. The employment of ether does not retard the healing of wounds nor give them an unfavorable character.

12th. The pains of death may often be relieved by etherization.

